# Acromioclavicular Joint Fixation Using an Acroplate Combined With a Coracoclavicular Screw

**DOI:** 10.5812/atr.10338

**Published:** 2013-06-01

**Authors:** Reza Tavakoli Darestani, Arash Ghaffari, Mehrdad Hosseinpour

**Affiliations:** 1 Orthopedics Surgery Department, Beheshti University of Medical Sciences, Tehran, IR Iran; 2 Trauma Research Center, Kashan University of Medical Sciences, Kashan, IR Iran

**Keywords:** Acromioclavicular Joint, Acroplate, Bone Screw

## Abstract

**Background:**

Appropriate treatment of acromioclavicular joint dislocation is controversial. Acroplate fixation is one of the most common treatment methods of acromioclavicular joint (ACJ) dislocation. Based on the risk of re-dislocation after Acroplate fixation, we assumed that combined fixation with an Acroplate and a coracoclavicular screw helps improve the outcome.

**Objectives:**

The main purpose of the current study was to compare the outcome of ACJ dislocation treated with an Acroplate alone and in combination with coracoclavicular screw.

**Patients and Methods:**

This study was carried out on 40 patients with ACJ dislocation types III to VI who were divided randomly into two equal groups: Acroplate group (P) and Acroplate in combination with coracoclavicular screw group (P + S). The screws were extracted 3-6 months postoperatively. The patients were followed for 1 year and Imatani’s score was calculated. Finally, the data were compared between the groups.

**Results:**

The mean Imatani’s score was significantly higher in P + S group (83.4 ± 14.1) than P group (81.2 ± 10.3) (P < 0.001). The mean duration of surgery was the same in the two groups (59.8 ± 9.4 minutes in group P V.s 64.3 ± 10.9 minutes in group P + S; P = 0.169). There were no cases of re-dislocation, degenerative changes and ossification and all patients returned to their previous jobs or sporting activities.

**Conclusions:**

Using a coracoclavicular screw combined with an Acroplate can improve the patients’ function after ACJ disruption without any significant increase in surgical duration. Authors recommend this technique in the fixation of ACJ dislocation.

## 1. Background

Acromiocalvicular Joint dislocation is a common injury responsible for 3 - 10% of shoulder girdle injuries (1, 2). For the patient to be able to return to the same level of previous activity, establishing the normal and primary anatomy of the joint is necessary (3). Different surgical approaches have been suggested for the treatment of acromioclavicular joint (ACJ) dislocation, including: coracoacromial ligament transfer, fixation with a plate, fixation with wire, screw fixation, coracoclavicular suture loop augmentation and new Tight Rope device, however, each method has its own deficiencies and complications (4-9). Although lots of surgical methods have been introduced for the treatment of ACJ dislocation, there are still controversies and the best fixation method is still unknown (5, 10-14). One of the traditional treatments in this kind of injury is using an Acroplate, which is a kind of hook plate. The outcome seems to be better if we use a Bosworth Screw concurrently because there is a chance of failure of the Acroplate.

## 2. Objectives

The aim of this study is to compare the outcome of ACJ dislocation treatment with an Acroplate alone and combined with a coracoclavicular screw.

## 3. Patients and Methods

**Figure 1.**
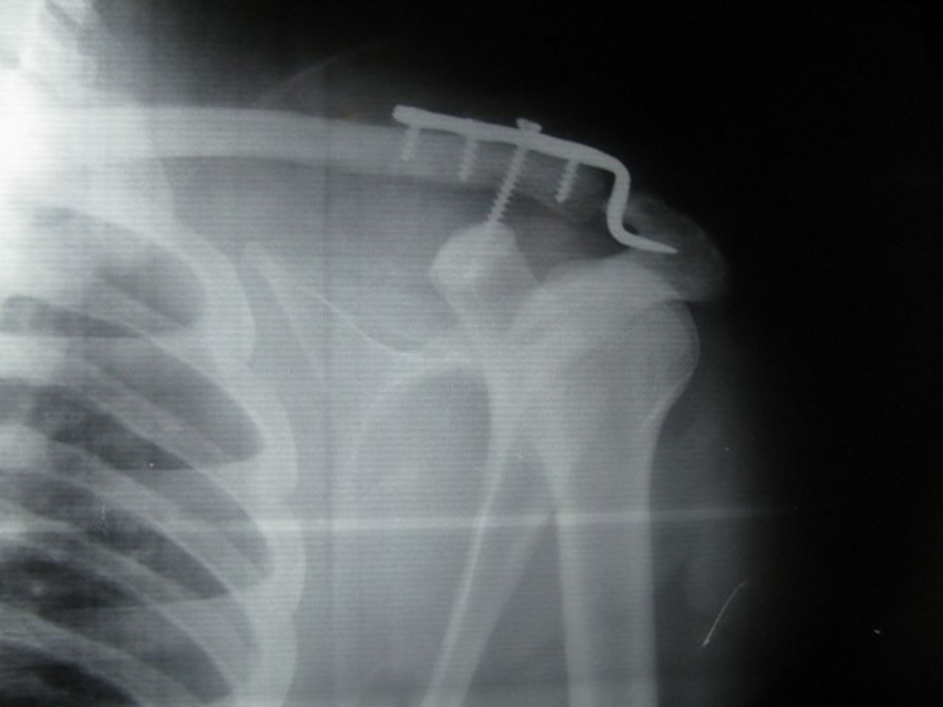
Acromioclavicular Joint Fixation Using an Acroplate

Between February 2011 and February 2012, 40 patients with grade 3 - 6 of Acromioclavicular Joint dislocation were referred to our hospital and were enrolled in our clinical trial. Patients were randomized into 2 groups: 20 had fixation with an Acroplate (P) and 20 had a coracoclavicular screw combined with an Acroplate (P + S). Patients' histories are presented in operating table and there was no significant statistical difference between the two groups. Informed consent was obtained from all participants who were eligible for our survey. It is important to know that the patients with grade III of acromioclavicular Joint dislocation were operated on only in the case of athletes or heavy workers. In group P after a standard linear incision and approach towards the AC Joint, the coracoclavicular and the acromioclavicular ligaments were reconstructed through direct repair and the joint was fixed with the plate. In group P + S we used a transverse incision in the distal part of clavicle over the AC Joint, and after reconstructing ligaments and fixation with an Acroplate, a semi-cancellous screw was inserted through the second hole of the plate, vertically into the coracoid process (Figure 1). As we were placing the screw, we checked its progression with the C-arm. After the operation, the extremity was immobilized with an abduction arm orthosis for 3 weeks. At the second postoperative day, the drain was discharged and passive external rotation exercises were initiated.

We removed the coracoclavicular screw under local anesthesia 3 - 6 months postoperatively. Patients were visited in 1, 2, 6 and 12 months follow-ups postoperatively and underwent accurate radiographic and physical examination. In the final visit the function of the injured shoulder was assessed by the Imatani’s scoring system which includes pain assessment, patient assessment and shoulder motion evaluation. Imatani’s scoring system is a functional and applicable scoring system and its easy to use and doesn’t take time. In postoperative radiographic examinations the joint direction was evaluated and any surgical complication was documented. In addition, in the final visit the patients were asked if they had the previous functional level of the shoulder to do their jobs or previous exercises (15).

## 4. Results

Imatani’s scoring system showed that the treatment outcome was not poor in either group. The average Imatani’s score in group P was 81.2 ± 10.3 (65 - 96) and in group P + S was 83.4 ± 14.1 (65 - 98). T-test revealed a significant statistical difference between the two groups, and group P+S had better outcomes (P < 0.001). The average surgery duration was 59.8 ± 9.4 minutes in group P and 64.3 ± 10.9 in group P + S. T test revealed no significant statistical difference between the two groups (P = 0.169). No ACJ re-dislocation was reported during the one year follow-up and in radiographic investigations, joint direction was normal. There was a case in group P + S with a screw fracture which led to an earlier screw removal, yet there were no other complications and specifically no ACJ dislocation. No degenerative changes or erosions were seen in the joint. In the final visit, all patients in both groups reported they had begun their previous jobs or exercises with no problems.

## 5. Discussion

Although treatment of ACJ dislocation with an Acroplate could result in good outcomes, the aim of this survey was to investigate whether coracoclavicular screw fixation combined with an Acroplate leads to a better outcome and joint function in the short term improving the patient's ability to do activities of daily living. In addition, the duration of surgery in group P+S was the same as group P and no meaningful difference was seen, although there was the further task of placing the coracoclavicular screw in group P + S. Even though more than 60 surgical methods for treatment of ACJ dislocation have been described, there is still no standard method for treatment of this injury (16-20). There are lots of controversies regarding the best treatment for ACJ dislocation to the extent that Smith et al. in a meta-analysis suggested that with the available surveys it’s impossible to discuss the preferences of surgical methods versus non-surgical methods (20). These treatment methods are generally classified into 5 groups including stabilizing ACJ, stabilizing the joint between coracoid and clavicle, dynamic muscle transfer, ligament reconstruction and distal clavicular resection (5, 10-14, 21). ACJ fixation is done with screws, k-wires or an Acroplate. Coracoclavicular joint stabilization first explained by Bosworth (22) is done with screws, synthetic objects or cerclage wires. Acroplates are associated with a firm and strong ACJ fixation (23). They don't interfere with ligament healing and help early motion of the joint. Previous surveys revealed that there is no complication associated with this method and the joint function will be well maintained; these features made this method the treatment of choice in ACJ dislocations (24). Di Feracesco et al. in 2012 treated 42 patients with ACJ dislocation using an Acroplate and assessed treatment outcomes by radiography and clinical tests. The plate was removed 3 months after the operation. Patients were visited postoperatively in the first and the third month (before plate removal) and also 1 year post-operatively and 18 months after surgery. They were evaluated by the Constant-Murley criteria. The researchers found that the incidence of re-dislocation after plate removal was 12% (5 patients). While MRI showed that in the remaining patients, coracoclavicular ligament had healed satisfactorily. The average constant score for patients with type 3 dislocations was 91.7 and 91.8 in patients with type 5 dislocations. Finally, the researchers concluded that using a Hook Plate for the treatment of ACJ dislocation was a good method and had desirable results in midterm follow-ups (25). Mechanical function of the coracoclavicular screw is nearly similar to the main ligaments (26). Screw loosening and re-dislocation are the most frequent complications of screw fixations (27). There are also reports of inflammation and infection (19). The biggest dilemma for using a coracoclavicular screw is the added operation needed for removing it. Generally, it has been suggested the the screw should be removed 8 weeks postoperatively (14). In the current survey because we didn’t use the screw alone, there were no re-dislocations reported, but there was a case of screw fracture as described earlier. Esenyel et al. in 2010 treated 32 patients with different ACJ dislocation types (3-6, 28) by coracoclavicular ligament reconstruction and coracoclavicular fixation. They used cancellous screws for 30 patients and cortical screws for the other 2; these 2 patients had screw cut-outs which led to a re-dislocation. Screws were removed 8 weeks postoperatively under local anesthesia and patients were followed for an average of 3.1 years. Average constant score in the 30 patients without re-dislocations was 98. Treatment results in 86.7% was excellent, while in 13.3% was good. One patient had acromioclavicular subluxation but joint direction remained normal in the other patients. No degenerative changes were seen in the patients and all patients had painless range of motion. Finally, these researchers reported that due to the great results and limited complications and also due to the low incidence of arthritis, this method was appropriate for the treatment of ACJ dislocation and restoration of normal shoulder function (28). Bektaser et al. reported an 8.8% re-dislocation rate in patients treated by the Bosworth technique (29). In addition, loss of fixation was reported in 16% of patients (19, 30, 31). It is obvious that using a coracoclavicular screw combined with an Acroplate can help eliminate these complications and the plate continues to protect the fixation even if the screw fixation is lost. Although there have been lots of surveys to evaluate different methods used for the treatment of ACJ dislocation with a coracoclavicular screw or an Acroplate, as we know, this survey is the first to evaluate the use of a coracoclavicular screw combined with an Acroplate in the treatment of ACJ dislocation. In other words, the aim of this survey was to assess whether using a coracoclavicular screw combined with an Acroplate was associated with better function and shoulder firmness in comparison with using an Acroplate alone. Furthermore, the duration of operation didn't differ significantly and didn't cause any complications. The current study was limited with the small sample size which may affect the outcomes. We declare that in order to make better conclusions further studies are required to investigate more patients. Using a coracoclavicular screw combined with an Acroplate can improve patients’ function after ACJ disruption without any significant increases in surgical duration. Authors recommend this technique in the treatment of ACJ disruptions.
